# Medical Profession in Paris

**Published:** 1849-01

**Authors:** 


*Medical Profession in Paris.*—It is stated in one of the Journals that Dr. Chomel, who has so extensive a practice, was an entire day, lately, without a single patient to visit; and that M. Marjolin, who ordinarily coins money during his hours of consultation, has not received, on an average, four dollars a day during the last month. This may furnish some idea of the state of distress of practitioners of less note.—*Medical News.*

